# Intramuscular cavernous haemangioma of the triceps

**DOI:** 10.11604/pamj.2020.36.139.23494

**Published:** 2020-06-30

**Authors:** Nabil Dammak, Hassen Cheikh Rouhou, Issam Khalifa, Ines Haddad, Yadh Zitoun, Faouzi Abid

**Affiliations:** 1 Department of Orthopaedic Surgery, University of Monastir, Taher Sfar Hospital of Mahdia, Mahdia, Tunisia,; 2 Anatomy and Cytopathology, University of Monastir, Taher Sfar Hospital of Mahdia, Mahdia, Tunisia

**Keywords:** Cavernous haemangioma, intramuscular haemangioma, triceps, X-ray, MRI

## Abstract

A 16-year-old teenager presented himself with a swollen left elbow, with no associated vascular-nerve complications. The standard radiography was without abnormalities. The echography showed the presence of an oblong vascularized formation occupying the posterior part of the elbow. The magnetic resonance imaging (MRI) showed a hyper vascularized lesion developing at the expense of the brachial triceps muscle with an intermediate signal intensity on the sequences weighted in T1 and a hyper signal in T2. The anatomopathological study of the initial biopsy and of the tumor part concluded with a cavernous hemangioma. Although their origin is vascular, hemangiomas never metastasize and do not undergo malignant transformation. The treatment of symptomatic hemangioma consists of surgical excision.

## Introduction

Cavernous hemangioma is a benign tumor of vascular pediculated origin, common in the orbit and the liver [1], rare in the breast and the cranial vault [2], but exceptional at elbow level. Our research in the indexed literature highlights only two cases affecting the elbow: the first intraosseous at the expense of the proximal part of the ulna [3], the second intramuscular affecting the triceps [4].

## Patient and observation

**Figure 1:**
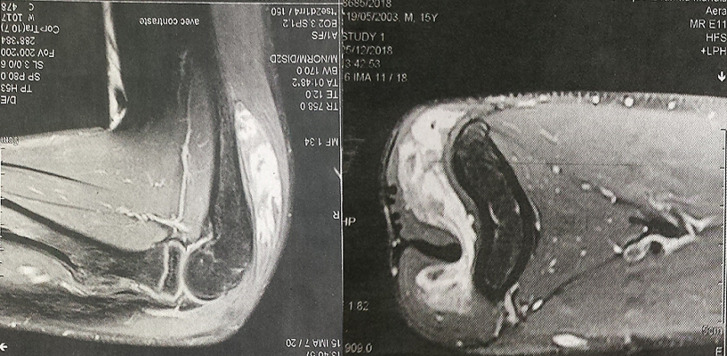
MRI of the elbow showing a hypervascularized mass depending on the triceps

**Figure 2:**
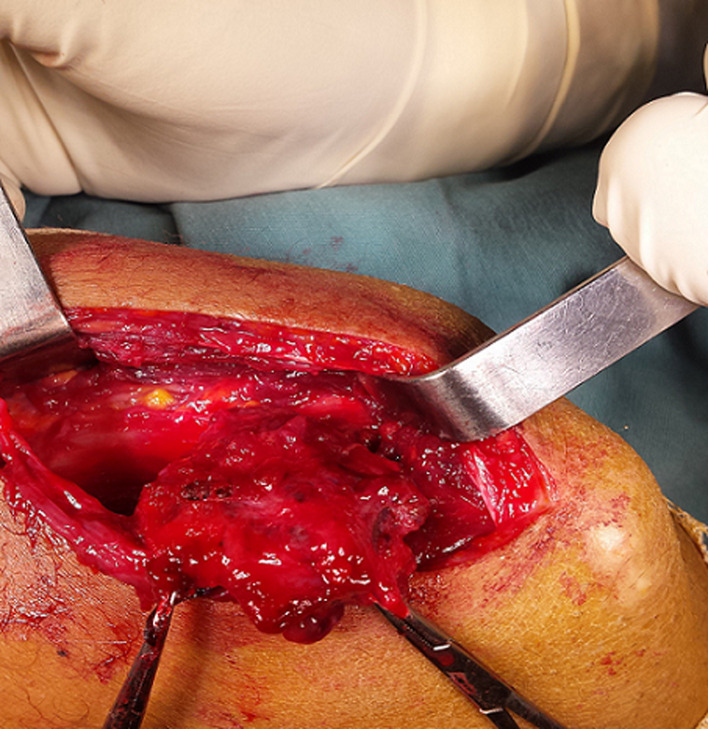
peroperative aspect: highly vascularized intramuscular tumor

**Figure 3:**
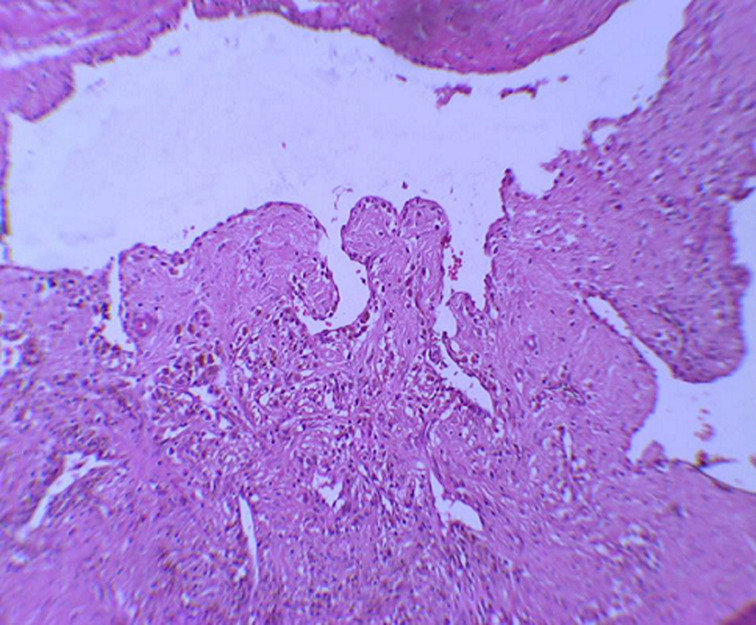
the lumen of some cavities is the site of intravascular papillary endothelial hyperplasia lesions (HEx20)

It is the case of a 16-year-old adolescent, with no particular medico-surgical history, who has been attended for one month by a practising orthopaedist for a swelling of the posterior aspect of the left elbow, appearing for 4 months and rapidly increasing in volume. He was asked for a standard X-ray showing no bone abnormalities, an echography that revealed the presence of an oblong formation occupying the posterior part of the vascularized elbow. Further exploration of MRI showed a hyper vascularised lesion developing at the expense of the brachial triceps muscle, measuring 75 x 70 x 16 mm, unrelated to the ulnar nerve (Figure 1). The youngster consulted us with his parents, fifteen days after having benefited from a first biopsy, performed in town, with an anatomopathological study concluding with a cavernous hemangioma. Our clinical examination found a scar from a postero-external approach to the left elbow; initial biopsy route; and demonstrated the underlying swelling occupying the entire posterior surface of the distal part of the arm, of firm consistency, painful on palpation, fixed with regard to the two plans and without local inflammatory signs or peripheral venous circulation. No vascular or nerve complications were recorded, particularly for the ulnar nerve. A surgical revision was performed to remove the tumour through the same approach of the biopsy: progressive dissection of the lesion and its entire resection (Figure 2). The normal ulnar nerve was distant from the tumour. We ended the intervention with a correct hemostasis, a rinse with physiological saline and a subcutaneous and cutaneous closure on a redon's suction drain. The tumor piece was entirely sent for anatomopathological study. The postoperative care was simple with the drain removal the day after the operation. The anatomopathological result maintained the initial diagnosis of cavernous haemangioma (Figure 3). Self-rehabilitation of the elbow was practiced with rapid restoration of its full function. Currently, at 1-year post excision, our youngster is fine, keeps a clean scar, a complete function of his elbow with no clinical stigma of tumour recurrence.

## Discussion

Histologically, hemangiomas are classified into three types: cavernous composed of multiple and dilated vessels, capillary with smaller and arteriovenous vessels made of arteries and dysplastic veins [5]. A combination of the cavernous and capillary types is frequently reported. Hemangiomas are distinguished from vascular malformations, clinically by rapid growth of the tumor volume during childhood and histologically by endothelial hyperplasia with the formation of a basement membrane laminated under the endothelium [6]. Intramuscular hemangiomas represent only 0.8% of all hemangiomas and most often occur in young people under the age of 30 in 80 to 90% of cases. The clinical examination did not find any specific characteristics for the swelling, but it was the pain which constitutes the cardinal symptom, present in 60% of cases [7]. The muscles of the lower limbs are more affected than those of the upper limbs with a predilection for the quadriceps [8]. Intramuscular hemangiomas gradually grow but no longer metastasize [9]. Their surgical excision leaves a recurrence rate of 9% [7]. For patients in whom excision is impractical, embolization or radiotherapy should be considered. In case of recurrence, adjuvant therapy (interferon alpha) may be considered. Our research in the indexed literature finds a single case of tricipital cavernous hemangioma with an ulnar nerve encapsulated by the tumour. In our patient, the nerve was normal in appearance, no longer having contact with the tumour. We also found another hemangioma case of the elbow region, but intraosseous affecting the proximal part of the ulna [3], discovered in an 18-year-old girl. Indeed, hemangiomas can affect any bone. The vertebrae and the skull are the most affected (75% of reported cases), unlike the long bones which are the least affected. Hemangiomas of long bones usually affect the bones of the lower limbs and ribs.

## Conclusion

We emphasize on ensuring ulnar nerve safety during the surgical procedure of cavernous hemangioma and the anatomopathological analysis for diagnosis confirmation.
